# Supplementary material for: Proteolytic profiling of human plasma reveals an immunoactive complement C3 fragment
Source: EMBO J. 2025 Oct 27;44(24):7721–58. doi: 10.1038/s44318-025-00598-8 (PMC12706077; doi:10.1038/s44318-025-00598-8)
Supplement: Supplementary file 1 — Appendix [file 44318_2025_598_MOESM1_ESM.pdf]

Appendix: “Proteolytic profiling of human plasma reveals an immunoactive complement C3 fragment”

Fatih Demir, Elina Kovalenko, Moritz Lassé, Esben Svenningsen, Jens Magnus Bernth Jensen, Anja M. Billing, Kathrin Groeneveld, Arvid Hutzfeldt, Lars Nilges, João P.L. Guerra, Krzysztof Jakub Pietrzak-Lichwa, Yifan Tan, Elizabeth Colby, Annette Gudman Hansen, Naziia Kurmasheva, David Olagnier, Dongwoo Choi, Mika M. Richter, Sandra D. Laufer, Fabian Braun, Sally A. Johnson, Marcus Krüger, Tobias B. Huber, Elion Hoxha, Oliver M. Steinmetz, Ralf Mrowka, Simon Melderis, Moin A Saleem, Thomas Poulsen, Gregers Rom Andersen, Steffen Thiel, Anne Troldborg, Markus M. Rinschen

Table of Contents

Semi-specific N-termini searches in the DIA space.....3

1. Entrapment searches.....3
2. Titration experiments.....4
3. Normalization strategies.....4

Ancillary Methods.....6

1. Entrapment searches.....6
2. E.coli spike-in experiments.....6

References.....7

Appendix Figure S1 (S)HUNTER method comparison.....8

Appendix Figure S2 Protein N-termini age in $^{13}C_6$-Lysine fed mice.....10

Appendix Figure S3 C3-LHF1 pull-down from plasma.....11

Appendix Figure S5 C3-LHF1 effect on HEK-Blue IL6Rα/IL6ST reporter line.....13

Appendix Figure S6 proteolySee resource.....14

Appendix Figure S7 Entrapment FDR.....15

Appendix Figure S8 E.coli Spike-in Titration Overview.....16

Appendix Figure S9 E.coli Spike-in Titrations.....17

Appendix Figure S10 N-termini coverage 19

Appendix Figure S11 Normalization & quantifications 20

Appendix Figure S12 N-termini coverage & quantification.. 21

Appendix Figure S13 N-termini coverage in CTRL / SLE.. 22

Appendix Figure S14 N-termini coverage and regulation 23

Semi-specific N-termini searches in the DIA space

While our paper was under review, a high-profile paper was published on controlling FDR in DIA datasets (Wen *et al*, 2025). This paper suggests that entrapment searches – assessing False detection positives (FDP) using different search strategies such as database shuffling, or foreign species searches, followed by various calculation strategies are necessary to control FDR; particularly protein FDR were shown to be strongly affected, especially in single-cell proteomics datasets.

Of course, the continuous development and check of protein and PSM FDR is an important issue. The paper, and also the previous preprint (doi: 10.1038/s41592-025-02719-x) has sparked active discussions in the field, demonstrating increased focus on the continuous development of algorithms, and control of FDR (e.g., https://github.com/Noble-Lab/FDRBench/issues/6#issuecomment-2987327065 or https://github.com/vdemichev/DiaNN/discussions/1035). Our laboratory relies on the excellent scholars benchmarking and adjusting search algorithms for FDR control. Currently, however, we cannot see a "gold standard" strategy on how best benchmark FDR in DIA data yet.

Our datasets are chiefly based on peptide PSM and the doubts about FDR control on the PSM level are relatively less compared to protein FDR. Yet, the semi-specific searches in DIA might pose an issue in FragPipe/DIA-NN analysis. To this end, we performed the following experiments and analysis to further address the issue:

1. Entrapment searches

We utilized the *combined and lower bound method* for FDP estimation as in (Wen *et al*, 2025). Therefore, we performed analyses using an Arabidopsis entrapment strategy to prove that our semi-specific free N-terminus identification strategy within FragPipe is robust. We applied differing peptide and PSM filtering thresholds within FragPipe utilizing three individual human plasma N-termini samples, queried against human canonical + isoforms, reviewed reference proteome database (42 525 entries) supplemented with the reviewed Arabidopsis reference proteome (18 643 entries, r

= 1.438 for combined database (Wen *et al*, 2025)). We communicated with the FragPipe developers and opted for the following settings for the search:

- Switching off percolator filtering (min. probability set to 0)
- Keeping protein level FDR fixed at 1% (not relevant for our peptide-level analysis)
- We altered peptide and PSM FDR within FragPipe from 1% to 10%

We could not observe a high entrapment FDP within the scopes or our evaluations (Appendix Figure S7), the determined "combined method" and "lower bound" FDP determinations were < 1% until the FDR cut-off was set to 5% (please see ancillary methods in the appendix for details).

2. Titration experiments

We performed titration experiments (Appendix Figure S8), where we spiked in E.coli protein into human plasma at different ratios (1:4, 1:2, 1:1, 2:1 and 4:1) and performed N-terminome analyses. We found that, without normalization, there was a clear increase in the total human N-termini intensity, but the nature of the identifications or the quantification of the human N-termini was not altered, suggesting that the identification is valid (Appendix Figure S8). In total, we believe that it is fair to say that semi-specific (N-terminomic) searches have a similar behavior as specific searches on the peptide FDR level, and identification and quantification is robust even in the presence of an excess of E.coli proteome.

3. Normalization strategies

To create a use case to see if the normalization may introduce biases, we compared the total DiaNN intensities per N-termini from human plasma with a variable amount of E.coli N-termini spiked in – with and without normalization. Despite the differing proteome ratios, we see that normalization is very effective and does not induce significant biases to a sample that has no spike-in (Appendix Figure S9). To assess the question, whether normalization based purely on post-enrichment abundance might introduce biases, particularly in samples with markedly different N-terminome coverage (e.g., 3% vs. 10% of all digested peptides), we calculated the mean protein N-termini sequence coverage as the peptide sequence coverage (Appendix Figure S10). We can observe a quite homogeneous distribution among the SLE cohort with a peak N-termini coverage of ~11% for all proteins per sample. For our

SHUNTERs we used the same protein amount for all samples (250ug per sample). That suggests that the total starting material is identical for all samples. Then, we performed the SHUNTER protocol and purified the N-termini. We observed that the peptide content in the N-termini-enriched samples post-enrichment did not differ among the control and SLE samples. In addition, biologically, we also do not see that normalization has an effect, at least on the alterations in SLE/CTRL in our patients (Appendix Figure S11).

Theoretically, it could remain an issue whether normalization was performed based on the total ion intensity pre- or post-enrichment. In our setting, we used the default settings of DiaNN which were used for all analyses. DiaNN performs a normalization step based on the ion total intensities post-enrichment (there are many protein-based normalization steps here, but they do not affect our data).

Investigating the individual protein N-termini coverage and the corresponding regulation of the N-terminus in SLE (Appendix Figure S12a) or the general abundance (as $\log_{10}$ intensity in Appendix Figure S12b) did not show any pronounced effect of the N-termini coverage on the interpretation for our N-termini data. Especially the regulation remains not affected by the individual protein N-termini coverage, whether DiaNN normalization was applied or not (Appendix Figure S12).

Comparing the CTRL and SLE samples in general, we could not observe general differences in the degree of N-termini coverage (Appendix Figure S13). Additionally, we inspected whether the distribution has any effect on the regulation in SLE by classifying the N-termini coverage into quantiles and comparing the quantifications (Appendix Figure S14). The $\log_2$FC(SLE/CTRL) was not statistically different.

In conclusion, it is important to consider that pre- or post-enrichment normalization might have an effect on the interpretability of N-termini data, but we believe that we have mitigated this issue by (1) using same amount of input material on proteome level (250 µg for all samples), and (2) showing that normalization does not have an overall effect on the biological effect of SLE in our patients, and (3) demonstrating that the N-termini coverage does not introduce a significant bias to the quantification.

Ancillary Methods

1. Entrapment searches

Three individual healthy human control EDTA-plasma samples were used to perform a SHUNTER N-termini enrichment with each 250 µg of starting proteome. Labeling, methodology and mass spectrometry were done exactly as for all the other SLE samples. Data analysis was performed with FragPipe v23.0 and the built-in DiaNN 1.8.1-beta2 and the following modifications to the normal data analysis workflow in the manuscript:

1. Percolator validation was switched off by setting the threshold to 0
2. For the validation within FragPipe, protein-level FDR was fixed to 0.01 (1%) as variations in the protein FDR had no influence on peptide-level identifications ("--prot 0.01")
3. Peptide and PSM-level FDR were varied from 0.01 (1%) to 0.1 (10%) by using the corresponding arguments ("--pep 0.01 --psm 0.01")
4. Total number of identified peptides and entrapped *Arabidopsis thaliana* peptides were counted from the peptides.tsv output file within FragPipe

2. E.coli spike-in experiments

In parallel to the three individual human EDTA-plasma samples, an E.coli cell pellet (0.48g Fw) was lysed in 2% NP-40, 50 mM HEPES pH 7.4, 0.1M NaCl, 2.5 mM EDTA, Roche cOmplete protease inhibitors for 1h at 4 °C and centrifuged at 13 000 g, 4 °C for 30 min. to pellet debris. The protein content in the lysate was determined via BCA and a big E.coli N-termini sample was generated in parallel to the human plasma samples. After N-termini enrichment, the peptide content of the purified N-termini was determined by NanoDrop A_{280} and corresponding total pools with 20 µg of N-termini and different spike-in E.coli N-termini amounts were generated in 1:4 to 4:1 ratio. From each mixture, 1 µg was injected into the Exploris480 nano-LC MS/MS mass spectrometer setup, measured and analyzed as for the SLE cohort samples. Data analysis was carried out with FragPipe v23.0 and the built-in DiaNN 1.8.1-beta2 with default settings for $^{13}CD_2O$-labelled N-termini (semi-specific free N-terminus, ArgC specificity, fixed dimethyl on K and peptide N-terminus and fixed carbamidomethyl on C; variable oxidation on M) and with switched off normalization ("--no-norm" option enabled).

References

Boisvert F-M, Ahmad Y, Gierliński M, Charrière F, Lamont D, Scott M, Barton G & Lamond AI (2012) A quantitative spatial proteomics analysis of proteome turnover in human cells. *Mol Cell Proteomics* 11: M111.011429

Lang F, Aravamudhan S, Nolte H, Türk C, Hölper S, Müller S, Günther S, Blaauw B, Braun T & Krüger M (2017) Dynamic changes in the mouse skeletal muscle proteome during denervation-induced atrophy. *Dis Model Mech* 10: 881–896

Li W, Dasgupta A, Yang K, Wang S, Hemandhar-Kumar N, Chepyala SR, Yarbro JM, Hu Z, Salovska B, Fornasiero EF, *et al* (2025) Turnover atlas of proteome and phosphoproteome across mouse tissues and brain regions. *Cell* 188: 2267-2287.e21

Rinschen MM, Gödel M, Grahammer F, Zschiedrich S, Helmstädter M, Kretz O, Zarei M, Braun DA, Dittrich S, Pahmeyer C, *et al* (2018) A Multi-layered Quantitative In Vivo Expression Atlas of the Podocyte Unravels Kidney Disease Candidate Genes. *Cell Rep* 23: 2495–2508

Tan Y, Chrysopoulou M & Rinschen MM (2023) Integrative physiology of lysine metabolites. *Physiol Genomics* 55: 579–586

Wen B, Freestone J, Riffle M, MacCoss MJ, Noble WS & Keich U (2025) Assessment of false discovery rate control in tandem mass spectrometry analysis using entrapment. *Nat Methods* 22: 1454–1463

Appendix Figure S1 (S)HUNTER method comparison

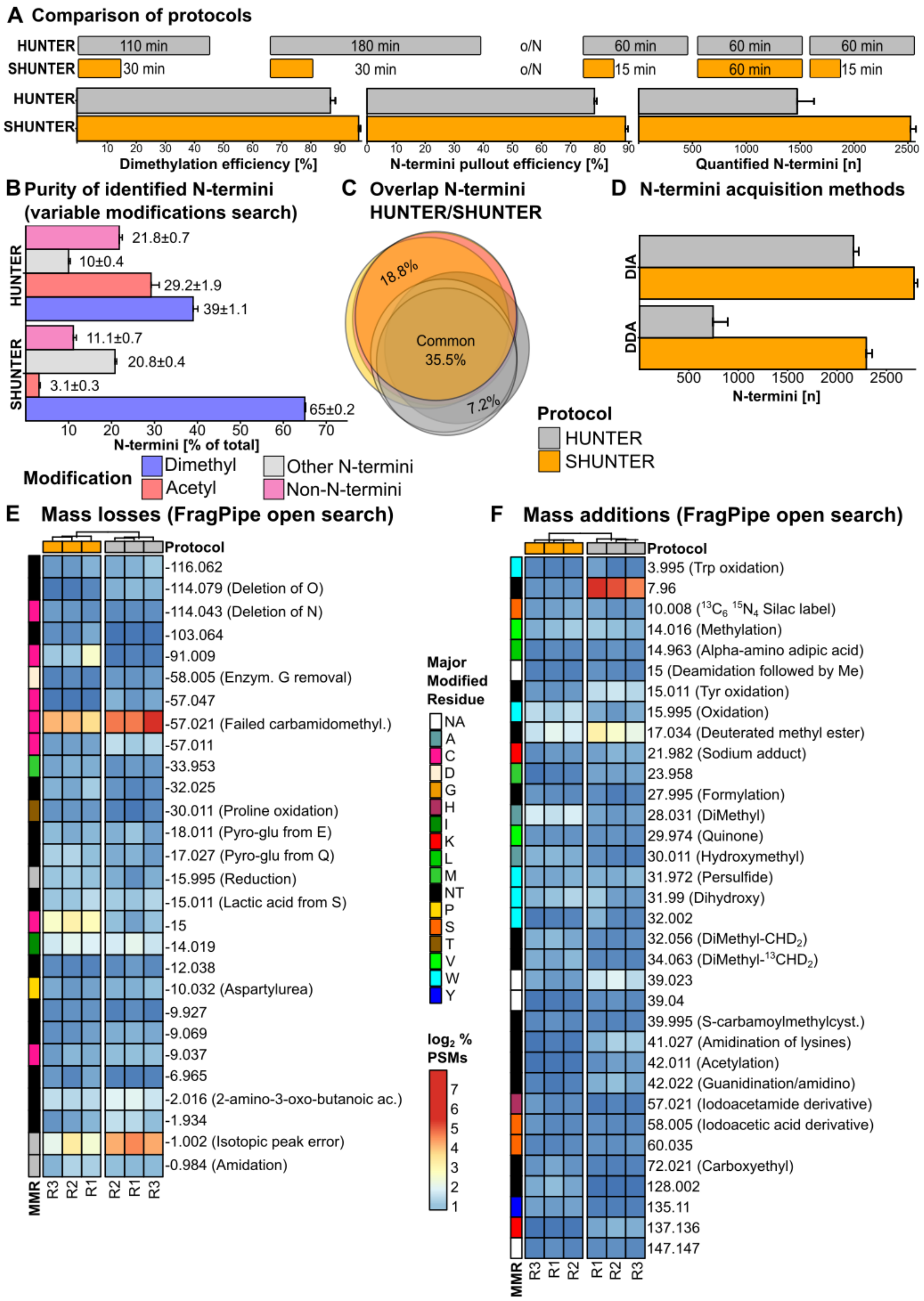

Appendix Figure S1: **Detailed overview of the differences between the workflows to access proteolytic events in human plasma. A**, A new, Shorter High-efficiency Undecanal-based N Termini EnRichment (SHUNTER) workflow was established. Dimethylation efficiency, N-termini purity (pullout efficiency), and quantified N-termini (n=3 technical replicates of human plasma, mean ± SE) are compared using the old HUNTER and the new SHUNTER workflow for N-termini enrichment from human plasma. **B**, Overlap of N-termini between different HUNTER/SHUNTER protocols from the same human plasma sample in all 3 methodological replicates (mean ± SE). Most N-termini are common to both protocols, but there are N-termini specific to the new or old protocol. **C**, Breakdown of N-termini by modification (n=3 technical replicates, mean ± SE). The dimethyl-modification fraction (blue) has markedly increased, and the degree of non-terminal contaminating tryptic peptides has been reduced (pink). **D**, Impact of acquisition method (data-independent DIA vs. data-dependent DDA) on N-termini identified illustrated the improvement in N-termini identifications, even with DDA (n=3 technical replicates, mean ± SE). **E&F**, Full open search profiles of peptide modifications using MSFragger's open search algorithms for both protocol variants. The $\log_2$ % PSMs of all total PSMs and the major modified residue (MMR) for each modification in each replicate (R, n=3 technical replicates) are shown.

Appendix Figure S2 Protein N-termini age in $^{13}C_6$-Lysine fed mice

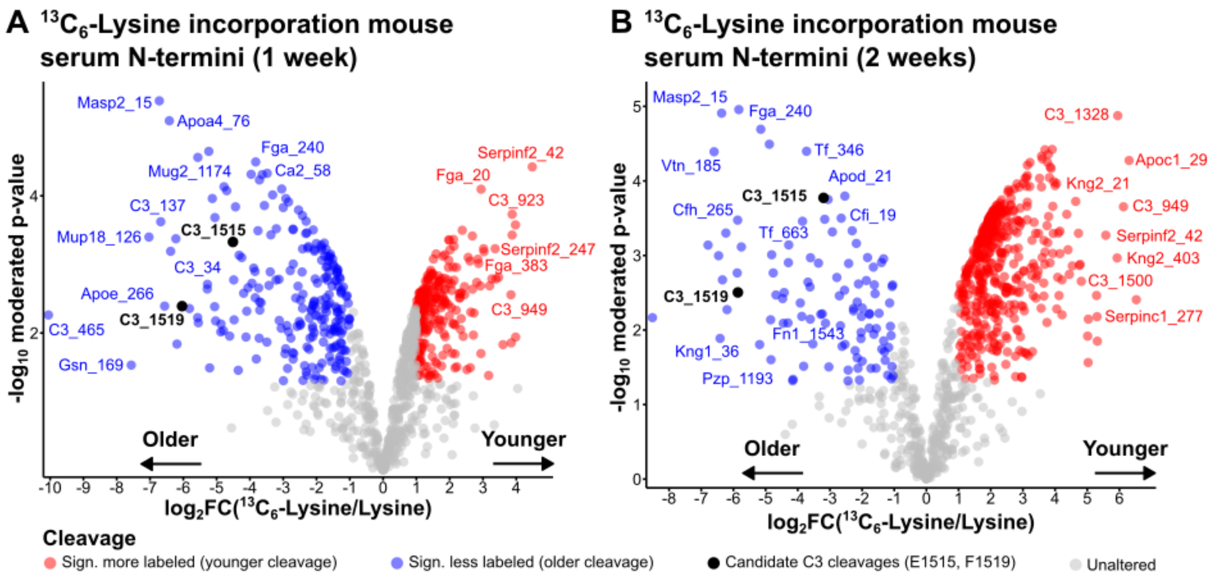

Appendix Figure S2: **Age of protein N-termini in $^{13}C_6$-Lysine fed mice**. Alterations ($|log_2FC| > 1$ & limma moderated t-test p-value < 0.05) in the stable-isotope labeled N-termini after in vivo isotope labeling with $^{13}C_6$-Lysine diet for 1 week (**A**, n=3 biological replicates) or 2 weeks (**B**, n=3 biological replicates). The cleavages C3_1515 and C3_1519 (marked in black) persist within the 2-week timeframe and show significantly less isotope labeling than other protein N-termini.

Appendix Figure S3 C3-LHF1 pull-down from plasma

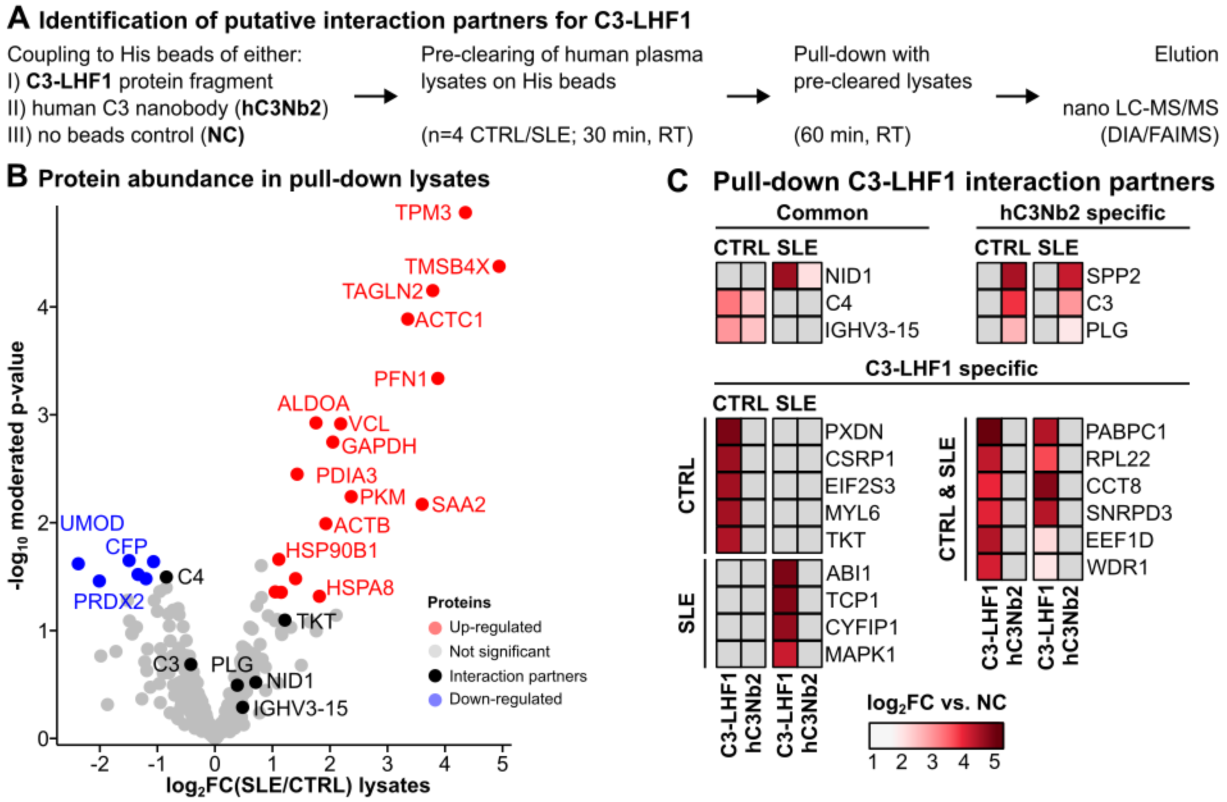

Appendix Figure S3: **C3-LHF1 pull-down from human plasma**. **A**, Workflow for identification of C3-LHF1 interaction partners from CTRL (n=4 individual samples) and SLE (n=4 patients) EDTA plasma samples. Lysates were pre-cleared on paramagnetic His purification beads and then equally split into three conditions: I) C3-LHF1, II) a nanobody against human C3 (hC3Nb2), and III) negative control (NC). Bound proteins were eluted with Lämmli buffer and subsequently processed with SP3 beads for proteome analysis. **B**, Global proteome profile for the exact used CTRL and SLE plasma samples revealed no significant alterations in protein abundance before C3-LHF1 pull-down for the identified proteins (n=4 biological replicates each, $|\log_2 FC > 1|$ & limma moderated t-test p-value < 0.05; significantly enriched proteins in pull-downs are marked in **black**). **C**, Significant targets ($\log_2 FC > 1$, limma moderates t-test p-value < 0.05, and quantification in 3 out of 4 replicates, no imputation) for hC3Nb2 and C3-LHF1 pull-downs are given. The C3 nanobody has a very narrow purification range, while C3-LHF1 purifies a greater number of proteins. The majority of the identified putative interaction partners are specific to CTRL or SLE plasma and can be categorized as cytoskeletal organization, signaling proteins, and members of the translation apparatus.

Appendix Figure S4 Assessment of bioactivity

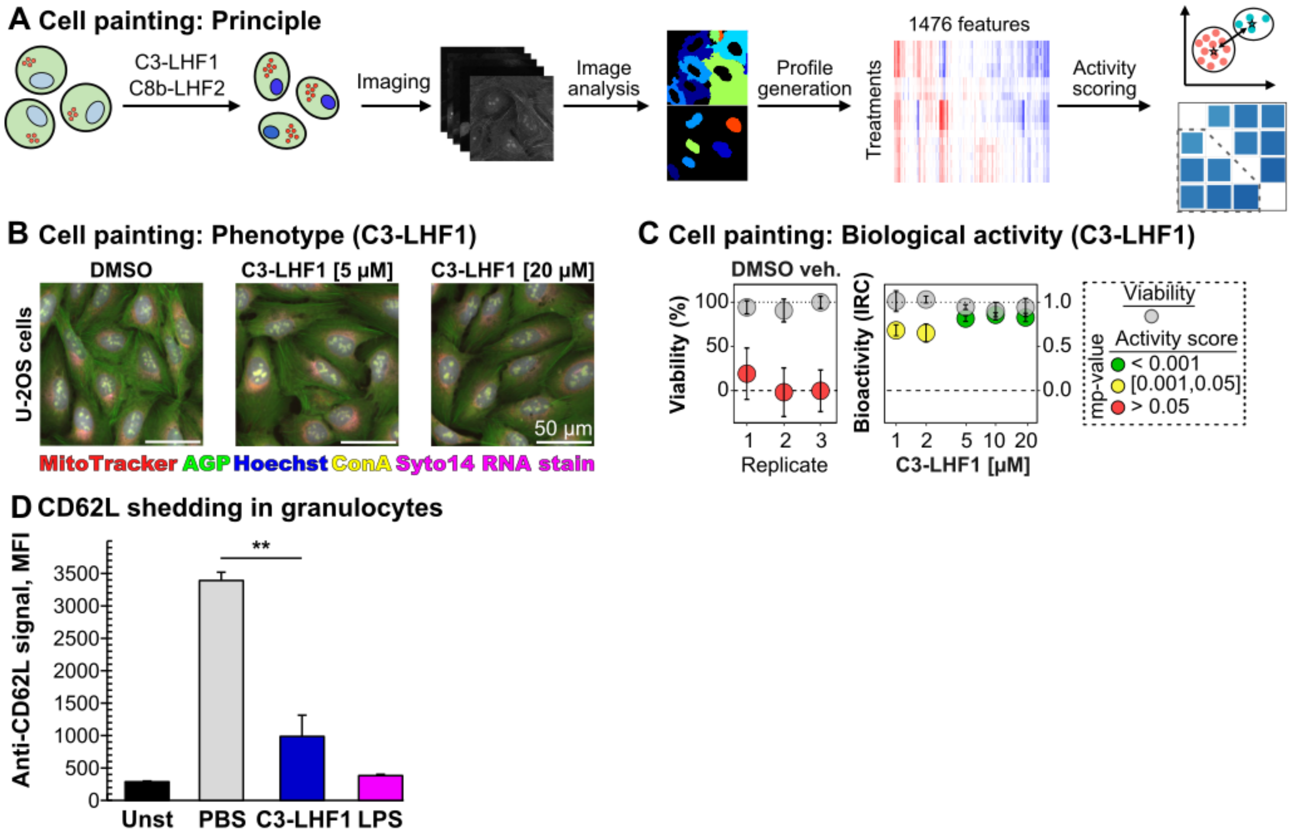

Appendix Figure S4: A, Overview of the cell painting technique to automatically assess biological activity and cell viability via multiplex staining. Generated cell painting profiles can be used to calculate activity and toxicity in a dose-dependent manner. **B,** Representative multiplexed fluorescent microscopy images from cell painting assay for C3-LHF1 display no substantial alteration in cell morphology (scale bar = 50 µm). **C**, Cytotoxicity profile of C3-LHF1 fragment reveals no change in viability but significant bioactivity of the compound at ≥ 5 µM compared to DMSO vehicle control (n=4 technical replicates, mean ± SD, multidimensional perturbation value (mp-value) < 0.001 defined as bioactivity). **D**, C3-LHF1 induces CD62L-shedding in granulocytes; the median fluorescence intensity (MFI) of detected CD62L on granulocytes (n=5 individual donors, mean ± SE; two-sided t-test p-value C3-LHF1 vs. PBS = $1.3x10^{-5}$, **) is illustrated for treatment with PBS, LPS at 0.19 mg/mL (pos. control), C3-LHF1 at 1.2 mg/mL. CD62L expression was detected using a fluorescent-labeled monoclonal antibody.

Appendix Figure S5 C3-LHF1 effect on HEK-Blue IL6Rα/IL6ST reporter line

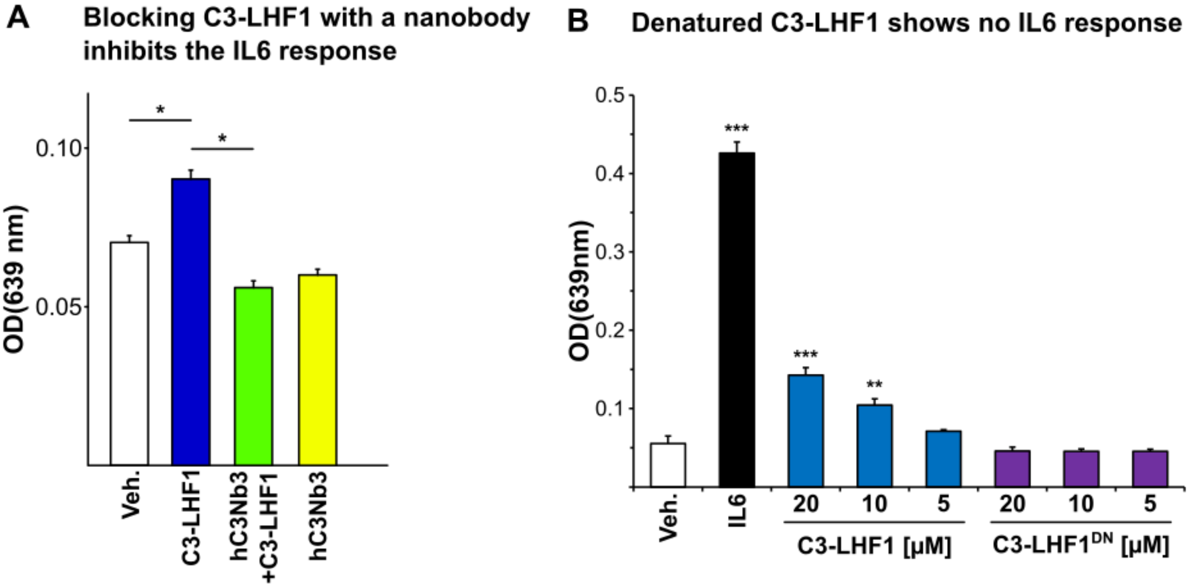

Appendix Figure S5: **Validation of C3-LHF1 effect on HEK-Blue IL6Rα/IL6ST reporter cell line.** **A**, Blocking C3-LHF1 with pre-incubation with the hC3Nb3 nanobody against the C345c domain of complement C3 (1:1 w/w ratio for 1h at 37 °C, C3-LHF1 concentration 5 µM) inhibits the IL6 response in the reporter line (n=4 technical replicates, mean ± SE; two-sided t-test p-value < 0.001 = *; p-value for C3-LHF1 vs. vehicle = $1.2x10^{-4}$, C3-LHF1 vs. hC3Nb3+C3-LHF1 = $7.5x10^{-4}$). **B**, Using denatured and carbamidomethylated C3-LHF1^{DN} does not evoke the same response as native C3-LHF1 at the tested concentrations of 20, 10 and 5 µM (n=4 technical replicates, mean ± SD; two-sided t-test p-value < 0.01 = **, < 0.001 = *** vs. vehicle control; IL6 used at $1x10^{-5}$ µg/mL; p-values for IL6 vs. vehicle = $1.3x10^{-7}$, 20 and 10 µM C3-LHF1 vs. vehicle = $2.9x10^{-5}$ and $5.7x10^{-4}$).

Appendix Figure S6 proteolySee resource

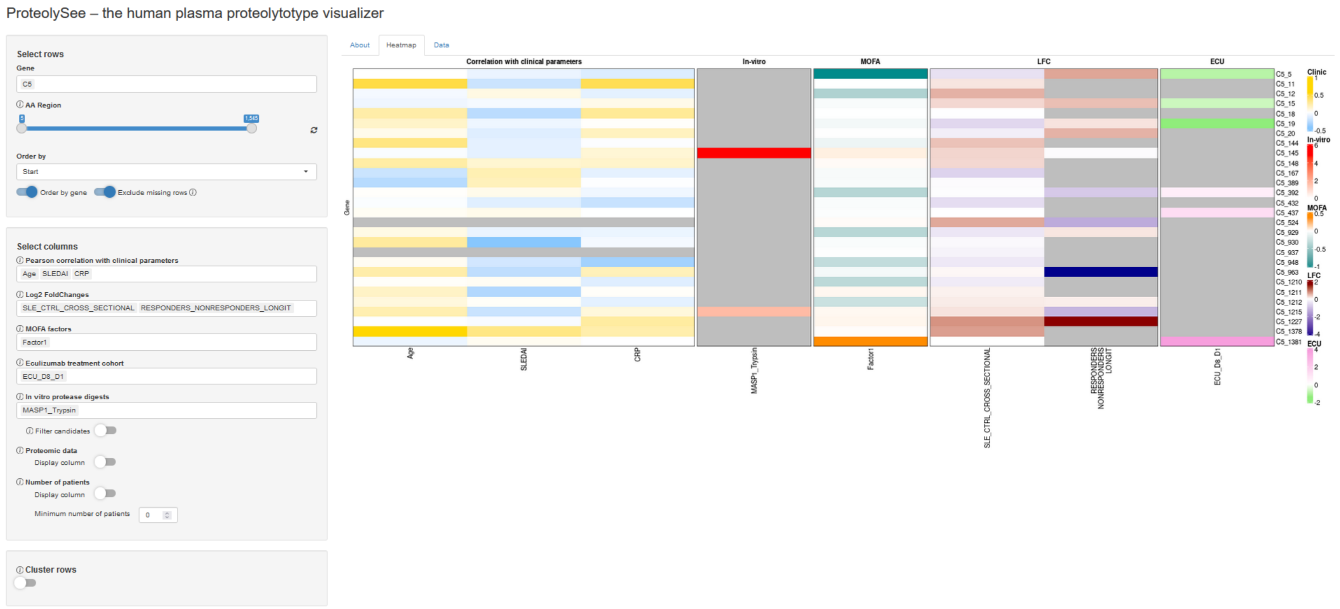

Appendix Figure S6: The proteolySee Resource to explore and mine the human proteolytotype. Screenshot of ProteolySee application, a human plasma proteolytotype visualizing app for aggregated data. The resource contains more than 10,000 termini from human plasma proteins, including their clinical associations and correlations, regulation in various conditions (lupus, lupus nephritis, Eculizumab treatment), as well as in vitro presence in the presence or absence of distinct proteases. Genes of interest, as well as regions of interest, can be selected. The website is accessible through https://ahutz.shinyapps.io/proteolysee/.

Appendix Figure S7 Entrapment FDR

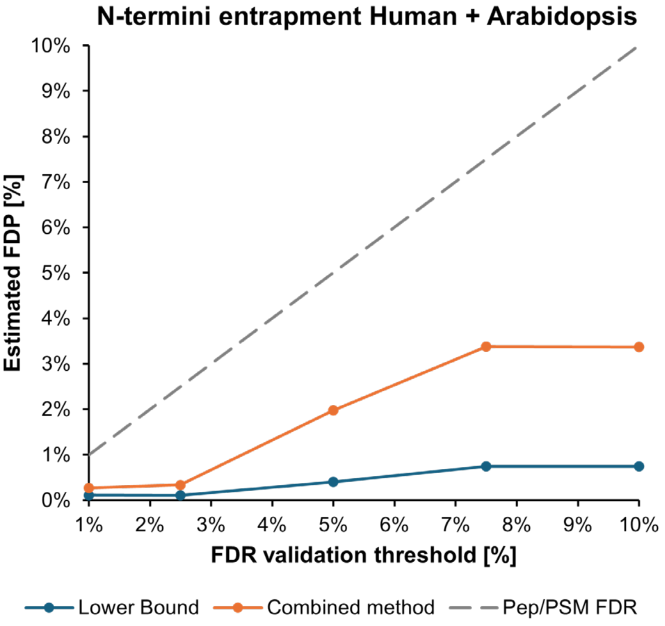

Appendix Figure S7: Entrapment searches (human + Arabidopsis thaliana) yielding a low estimated FDP for differing FDR cut-offs within semi-specific free N-terminus searches with FragPipe/DiaNN. With the usually applied 1% cut-off, the FDP is below 1% FDR – only after setting the peptide- and PSM-level FDRs to ≥ 5%, the level of bait identifications within Arabidopsis increases over the 1% FDP threshold.

Appendix Figure S8 E.coli Spike-in Titration Overview

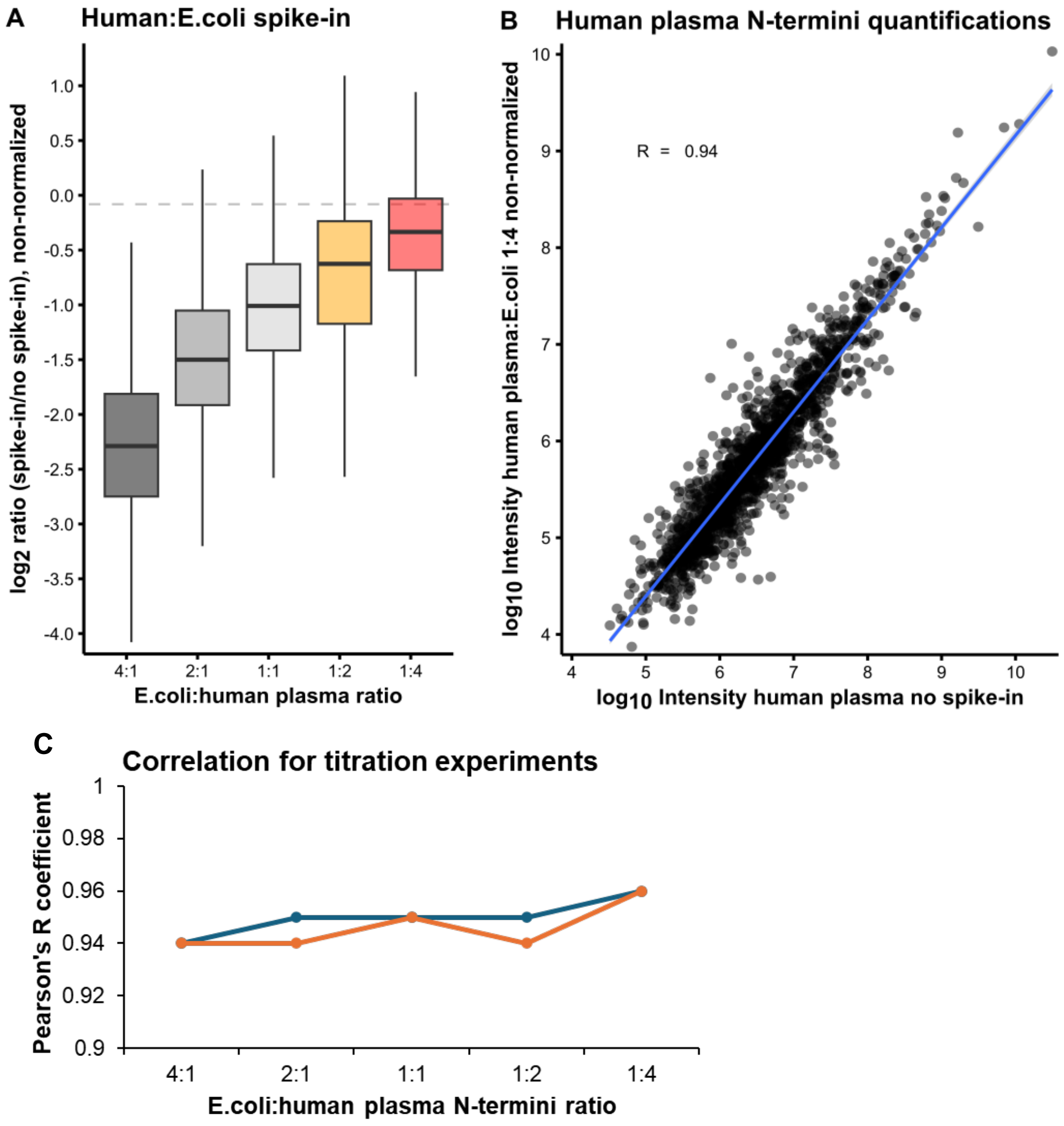

Appendix Figure S8: Robustness of the human N-termini quantifications with DiaNN/FragPipe were assayed with the help of human plasma samples (n=3 individual healthy controls) with alternating E.coli N-termini spike-in ratios (4:1 to 1:4 E.coli to human plasma). **A**, Assayed in a non-normalized manner with DiaNN, increasing E.coli spike-ins lead to reduced ratios comparing against the control. non-spiked control measurements. **B**, Comparing the DiaNN quantifications (normalized) for the human N-termini in the 4:1 E.coli:human plasma spike-in with the non-spiked human plasma reveals a robust quantification of the human N-termini regardless of a 4:1 spike-in of E.coli (Pearson's correlation coefficient of 0.94). **C**, Pearson's correlation coefficients for the 4:1 to 1:4 E.coli:human plasma N-termini spike-ins reveal a very high degree of correlation for the spike-in and no spike-in control sample quantifications for the human plasma N-termini.

Appendix Figure S9 E.coli Spike-in Titrations

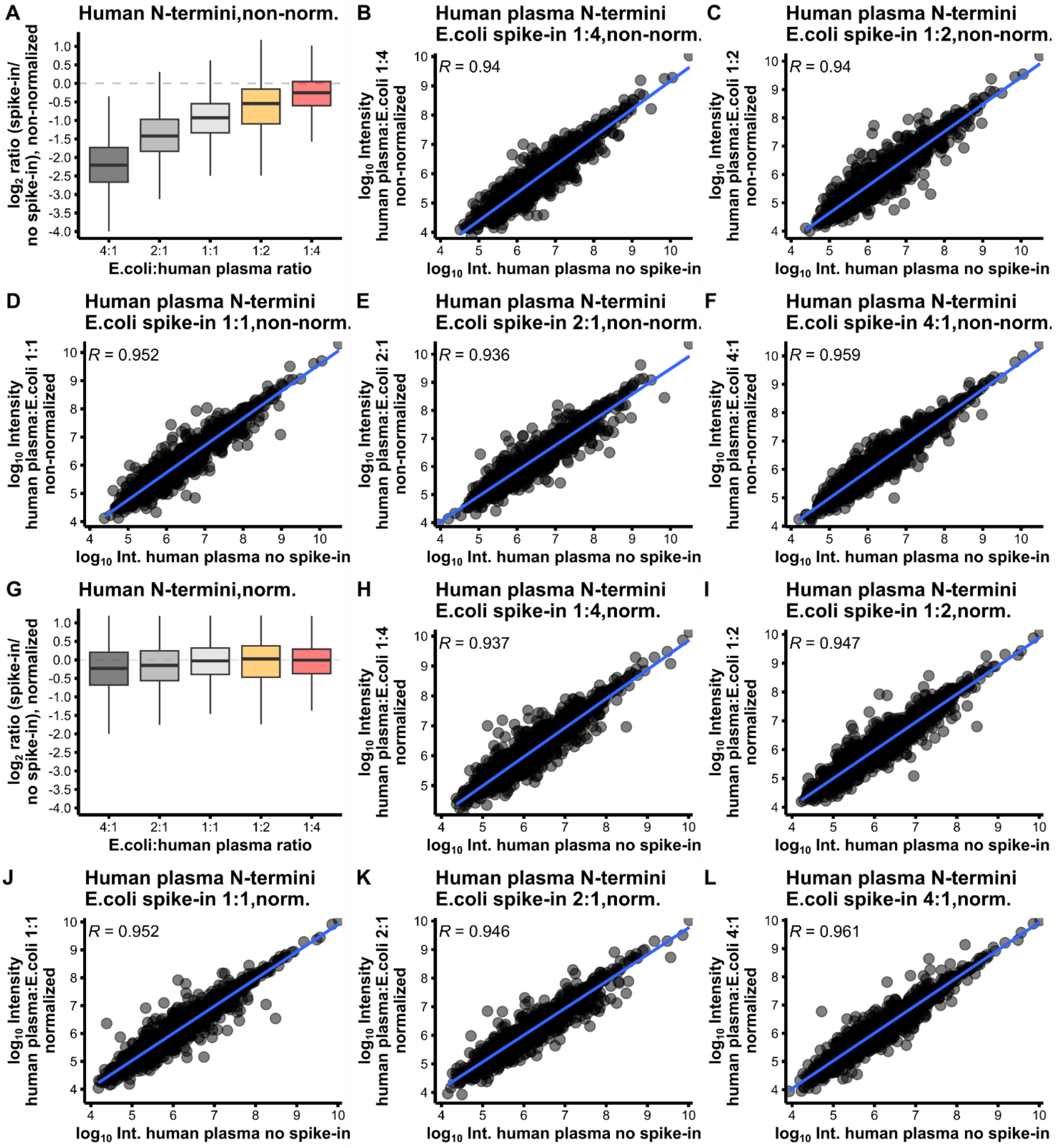

Appendix Figure S9: Robustness of human plasma N-termini quantifications (n=3 individual samples) with or without normalization during DiaNN quantification. E.coli spike-ins were performed at different ratios of 4:1 to 1:4 on peptide level. **A**, Distribution of human plasma N-termini quantification ratios against no E.coli spike-in control (non-normalized). **B**, Excess E.coli does not

affect quantification of the human plasma N-termini (ratio 1:4 human plasma:E.coli, non-normalized, Pearson's correlation coefficient R = 0.94). Decreasing amounts of E.coli do not alter the correlation of the N-termini quantifications (**C-E**), also at low amounts of E.coli (**F**, ratio 4:1, non-normalized, R = 0.959). **G**, Distribution of measurements, queried with DiaNN normalization displays the capabilities of DiaNN to normalize the human plasma N-termini quantifications even in higher E.coli spike-in ratios like 1:4 (**H**) or 1:2 (**I**). Quantifications are stable throughout the tested 1:1 (**J**), 1:2 (**K**), 1:4 (**H**) and 4:1 (**F**) ratios with a high degree of correlation (**H**, R = 0.937 for 1:4 and **I**, R = 0.961 for 4:1).

Appendix Figure S10 N-termini coverage

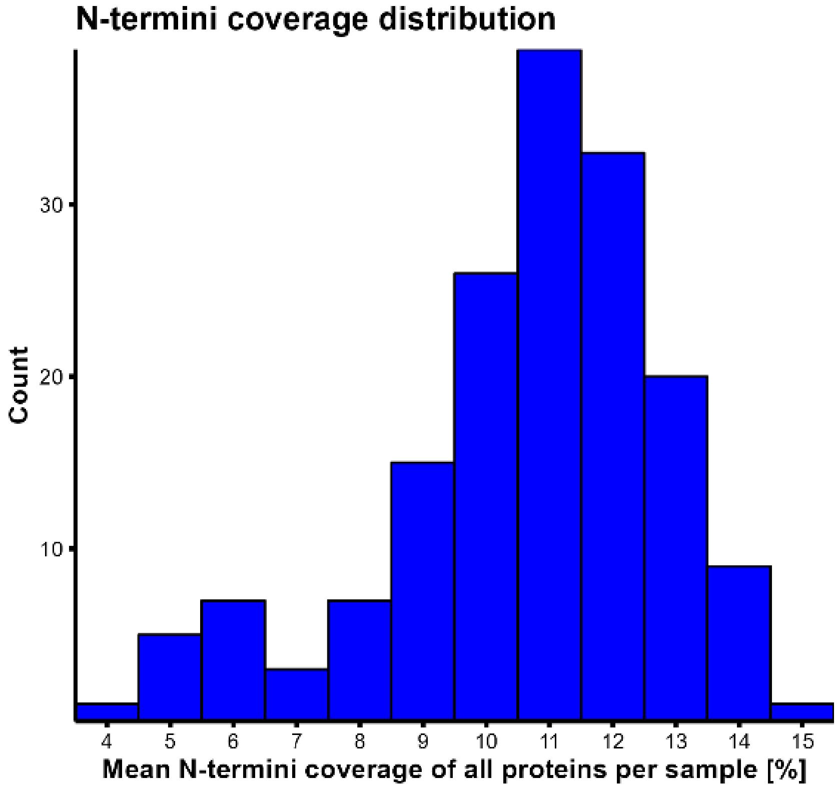

Appendix Figure S10: Overall mean N-termini coverage distribution for SLE cohort samples.

Appendix Figure S11 Normalization & quantifications

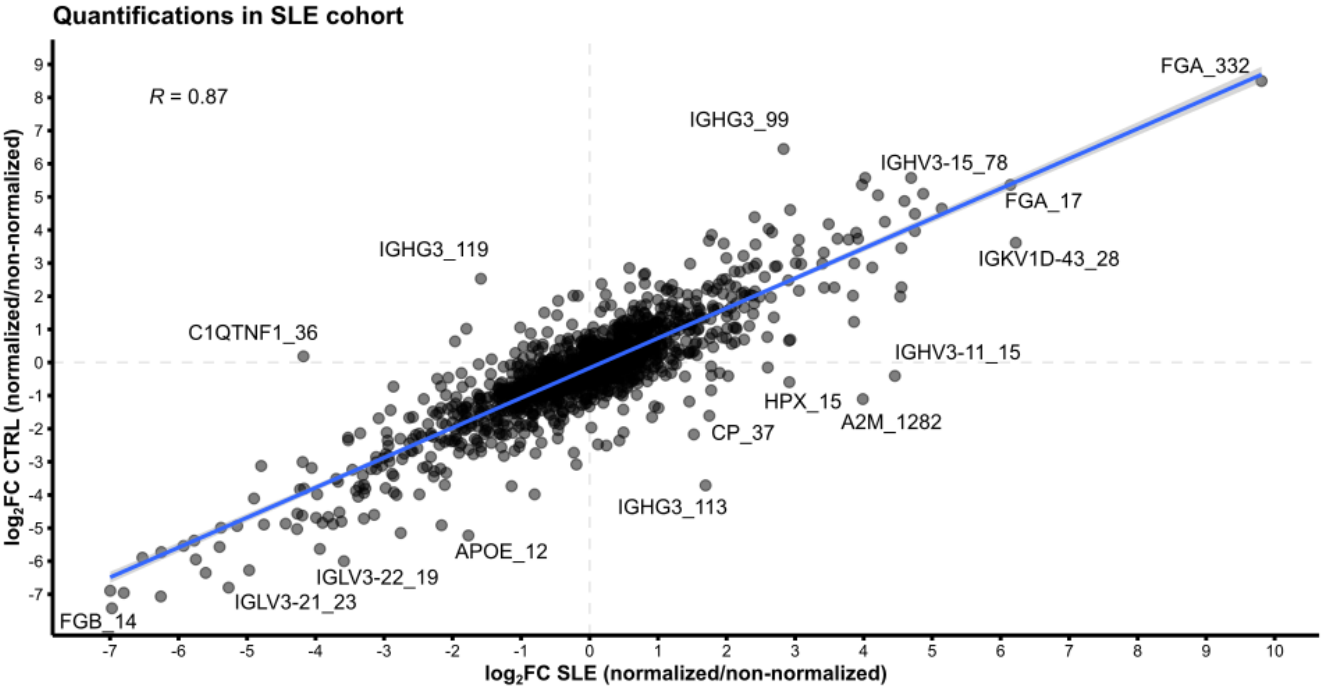

Appendix Figure S11: Regulation in the SLE and CTRL samples shows a high degree of correlation for normalized/non-normalized ratios, thus the effect of the DiaNN-inherent normalization has no substantial effect on the quantification.

Appendix Figure S12 N-termini coverage & quantification

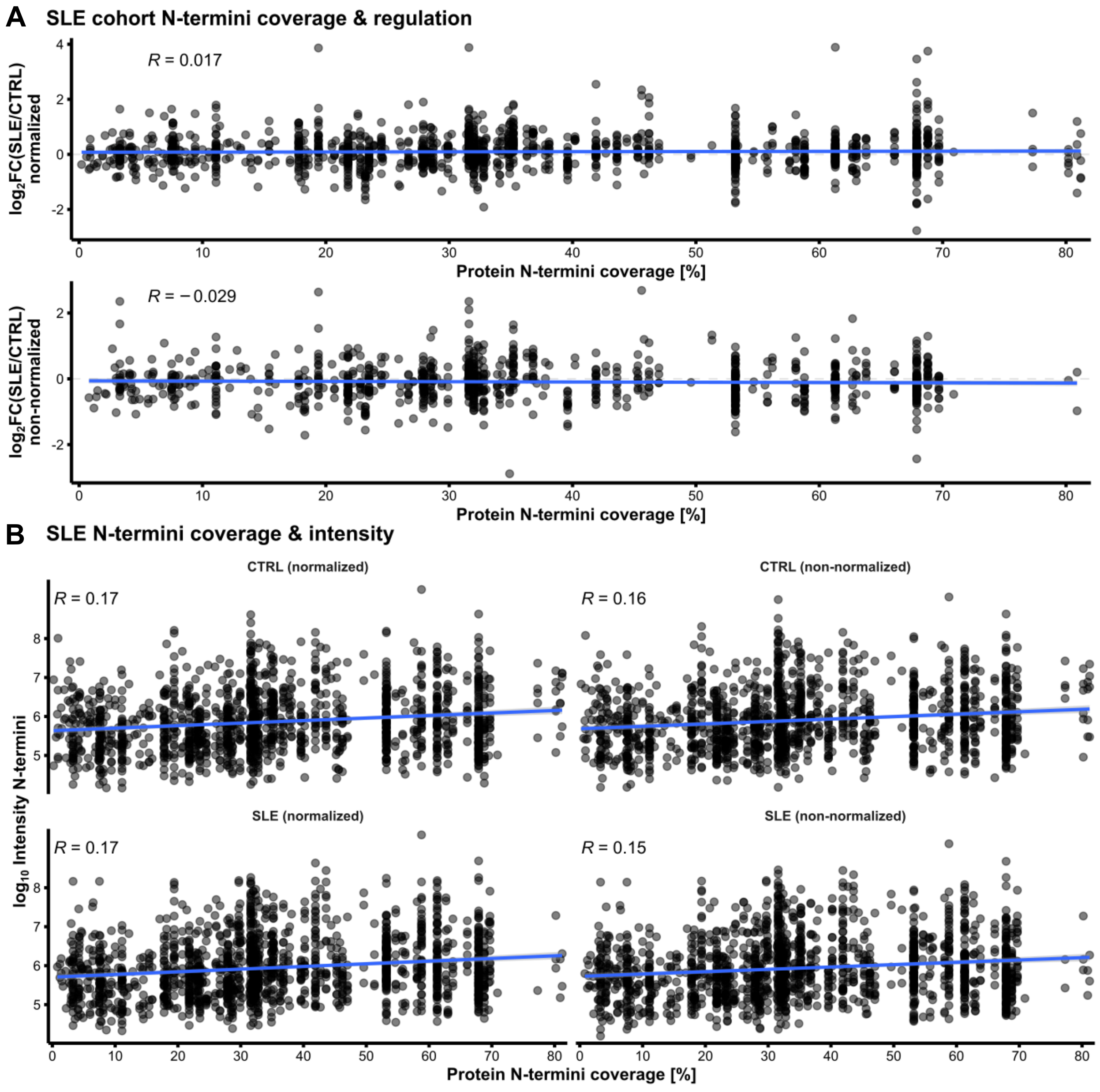

Appendix Figure S12: The protein N-termini coverage in the SLE cohort was mapped to the regulation and intensities. **A**, No correlation can be observed for the protein N-termini coverage with the regulation in log_2FC(SLE/CTRL) – regardless of if normalized or non-normalized. **B**, There is no correlation for the log_{10} intensities with the protein N-termini coverage, which is comparable among the CTRL and SLE samples and does not differ greatly among the normalized or non-normalized samples.

Appendix Figure S13 N-termini coverage in CTRL / SLE

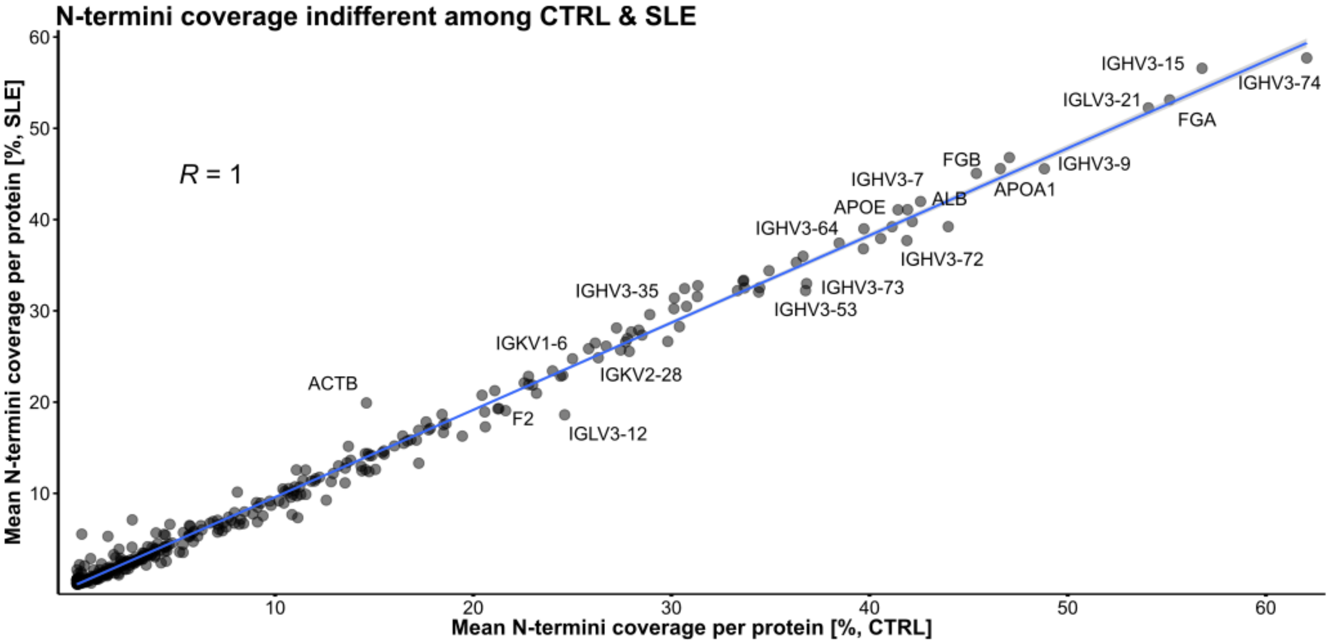

Appendix Figure S13: The mean protein N-termini coverage did not differ among the SLE and CTRL samples (Pearson's correlation coefficient of 1.0).

Appendix Figure S14 N-termini coverage and regulation

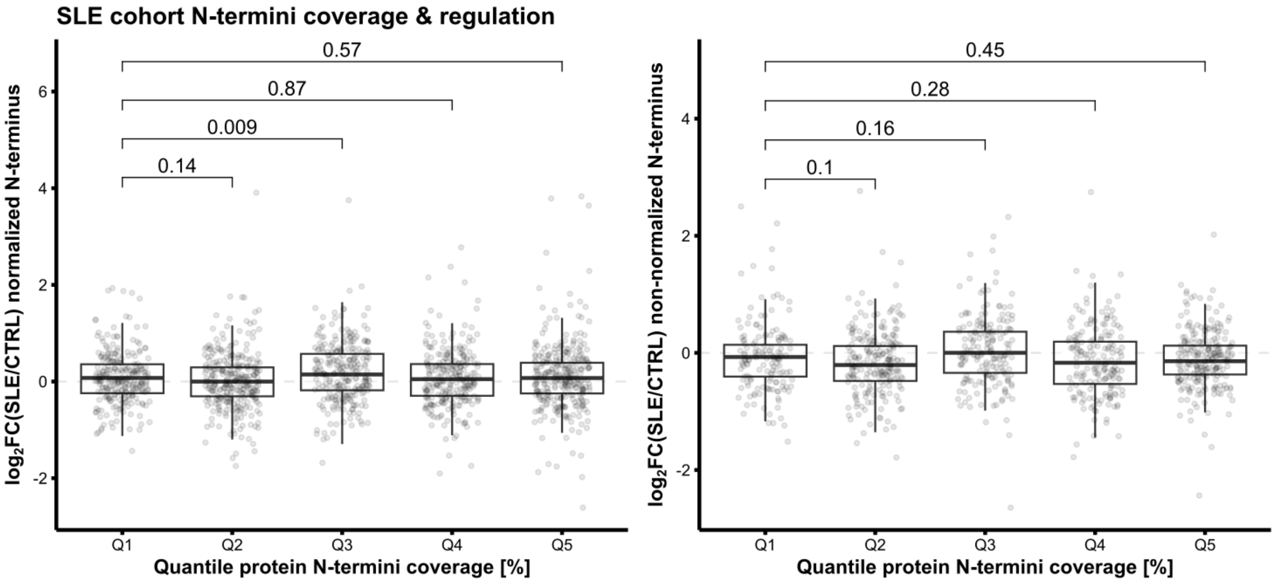

Appendix Figure S14: The protein N-termini coverage was classified into quantiles (Q1: 0-18%, Q2: 19-30%, Q3: 31-36%, Q4: 37-56%, Q5: 67-81%) and DiaNN N-termini quantifications were determined – no significant difference (two-sided t-test p-values are given) was observed between the quantiles, regardless if DiaNN N-termini quantifications were normalized (left) or not (right).
