# Supplementary material for: Proteolytic profiling of human plasma reveals an immunoactive complement C3 fragment
Source: EMBO J. 2025 Oct 27;44(24):7721–58. doi: 10.1038/s44318-025-00598-8 (PMC12706077; doi:10.1038/s44318-025-00598-8)
Supplement: Supplementary file 29 — Expanded View Figures [file 44318_2025_598_MOESM29_ESM.pdf]

Expanded View Figures

Figure EV1. Application of N-termini enrichment to complement inhibition through eculizumab.

(**A**) Patient plasma samples with Shiga Toxin producing *E. coli*-induced hemolytic uremic syndrome (STEC-HUS) were treated with anti-C5 antibody (eculizumab), and N-termini quantified after day 8 and day 30 of treatment, compared to baseline conditions. Comparison of N-terminome after day 8 (**B**), $n = 4$ patients) or day 30 (**C**), $n = 3$ patients) of eculizumab (anti-C5) treatment vs baseline (day 1). Significantly decreased and increased ($|\log_2 FC| > 1$ & limma moderated t-test *p*-value 0.05) N-termini are depicted in blue and red, correspondingly. Alterations in the N-terminome are more diverse and pronounced on day 8, whereas on day 30, hemopexin cleavages are increasingly present. The C5 N-terminus C5_1381 is significantly up-regulated at day 8 and substantially up-regulated at day 30 of eculizumab treatment (marked in black). (**D**) Scatterplot of the $\log_2$FC N-terminome vs. the $\log_2$FC of total proteome after 8 days or 30 days of treatment (**E**) of treatment. Red data points correspond to complement proteins and show a lower correlation between N-termini and proteome abundance. The corresponding correlation is given as Pearson's correlation coefficient (R) and the significance as paired t-test *p*-values. (**F**) Significantly regulated complement N-termini ($|\log_2 FC| > 1$ & limma moderated t-test p-value < 0.05) during Eculizumab treatment feature a strongly regulated N-terminus for C5 (C5_1381) after day 8 of eculizumab treatment. (**G**) Structure of complement C5 in complex with the Fab fragment of eculizumab binding to an epitope in the C5/C5b MG7 domain. Gray spheres correspond to the first and last residue of C5. An orange sphere indicates the position of Ile1381. Right, magnified view of the cleavage site proximal to flexible loop in the MG8 domain and the ANA domain released by C5 convertases. Cleavage at position 1381 may lead to partial unfolding of the MG8 domain.

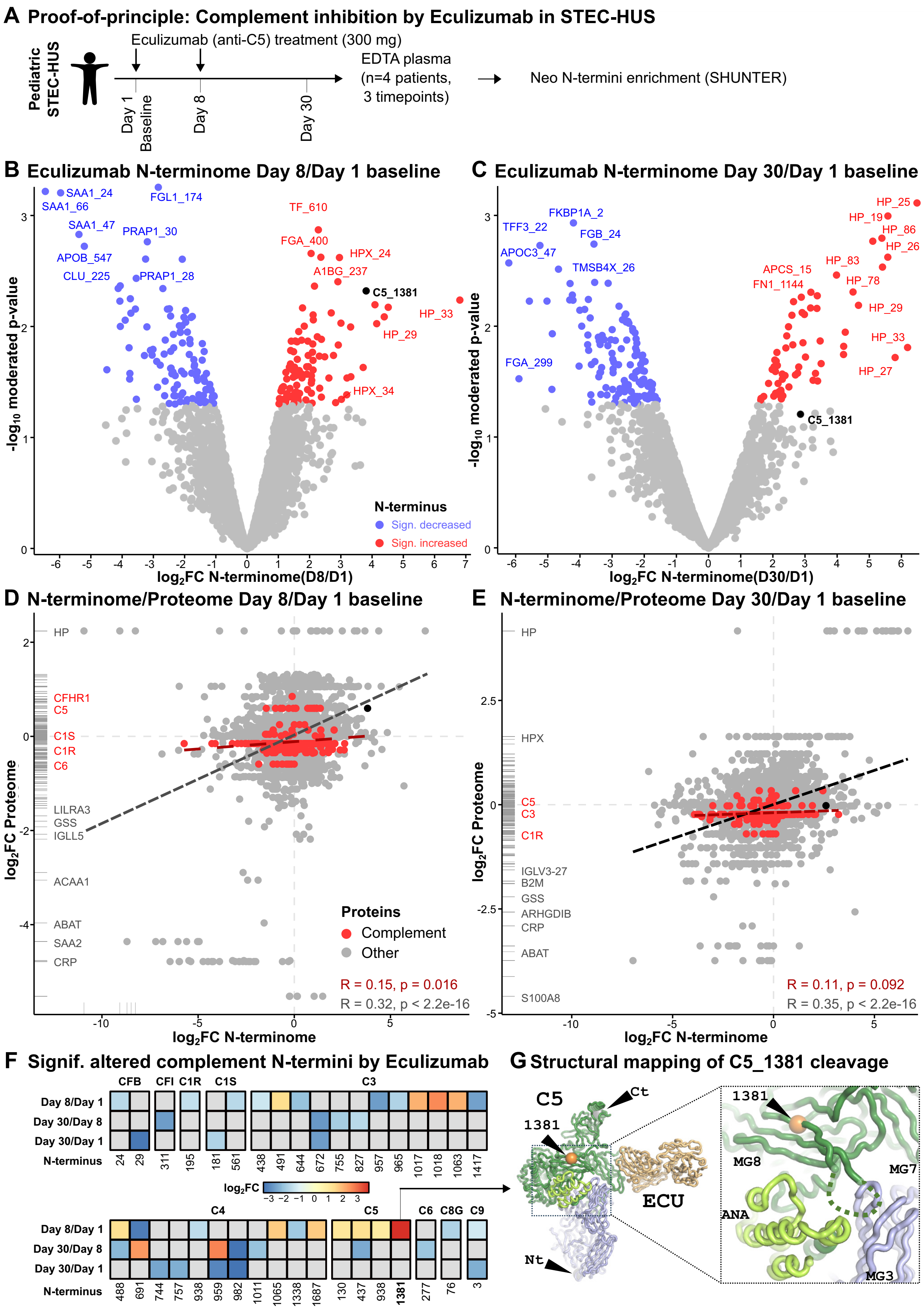
A Proof-of-principle: Complement inhibition by Eculizumab in STEC-HUS
Eculizumab (anti-C5) treatment (300 mg)
Pediatric STEC-HUS
Day 1 Baseline
Day 8
Day 30
EDTA plasma (n=4 patients, 3 timepoints)
Neo N-termini enrichment (SHUNTER)
B Eculizumab N-terminome Day 8/Day 1 baseline
C Eculizumab N-terminome Day 30/Day 1 baseline
-log10 moderated p-value
log2FC N-terminome(D8/D1)
log2FC N-terminome(D30/D1)
N-terminus
Sign. decreased
Sign. increased
SAA1_24
SAA1_66
FGL1_174
SAA1_47
PRAP1_30
APOB_547
CLU_225
PRAP1_28
TF_610
FGA_400
HPX_24
A1BG_237
C5_1381
HP_33
HP_29
HPX_34
FKBP1A_2
TFF3_22
FGB_24
APOC3_47
TMSB4X_26
FGA_299
HP_25
HP_19
HP_86
HP_26
HP_83
APCS_15
FN1_1144
HP_78
HP_29
HP_33
HP_27
D N-terminome/Proteome Day 8/Day 1 baseline
E N-terminome/Proteome Day 30/Day 1 baseline
log2FC Proteome
log2FC N-terminome
HP
CFHR1
C5
C1S
C1R
C6
LILRA3
GSS
IGLL5
ACAA1
ABAT
SAA2
CRP
Proteins
Complement
Other
R = 0.15, p = 0.016
R = 0.32, p < 2.2e-16
HPX
C5
C3
C1R
IGLV3-27
B2M
GSS
ARHGDIB
CRP
ABAT
S100A8
R = 0.11, p = 0.092
R = 0.35, p < 2.2e-16
F Signif. altered complement N-termini by Eculizumab
CFB CFI C1R C1S C3
Day 8/Day 1
Day 30/Day 8
Day 30/Day 1
N-terminus
24 29 311 195 181 561 438 491 644 672 755 827 957 965 1017 1018 1063 1417
log2FC
C4 C5 C6 C8G C9
488 691 744 757 938 959 982 1011 1065 1338 1687 130 437 938 1381 277 76 3
G Structural mapping of C5_1381 cleavage
C5
Ct
1381
ECU
Nt
MG8
MG7
ANA
MG3

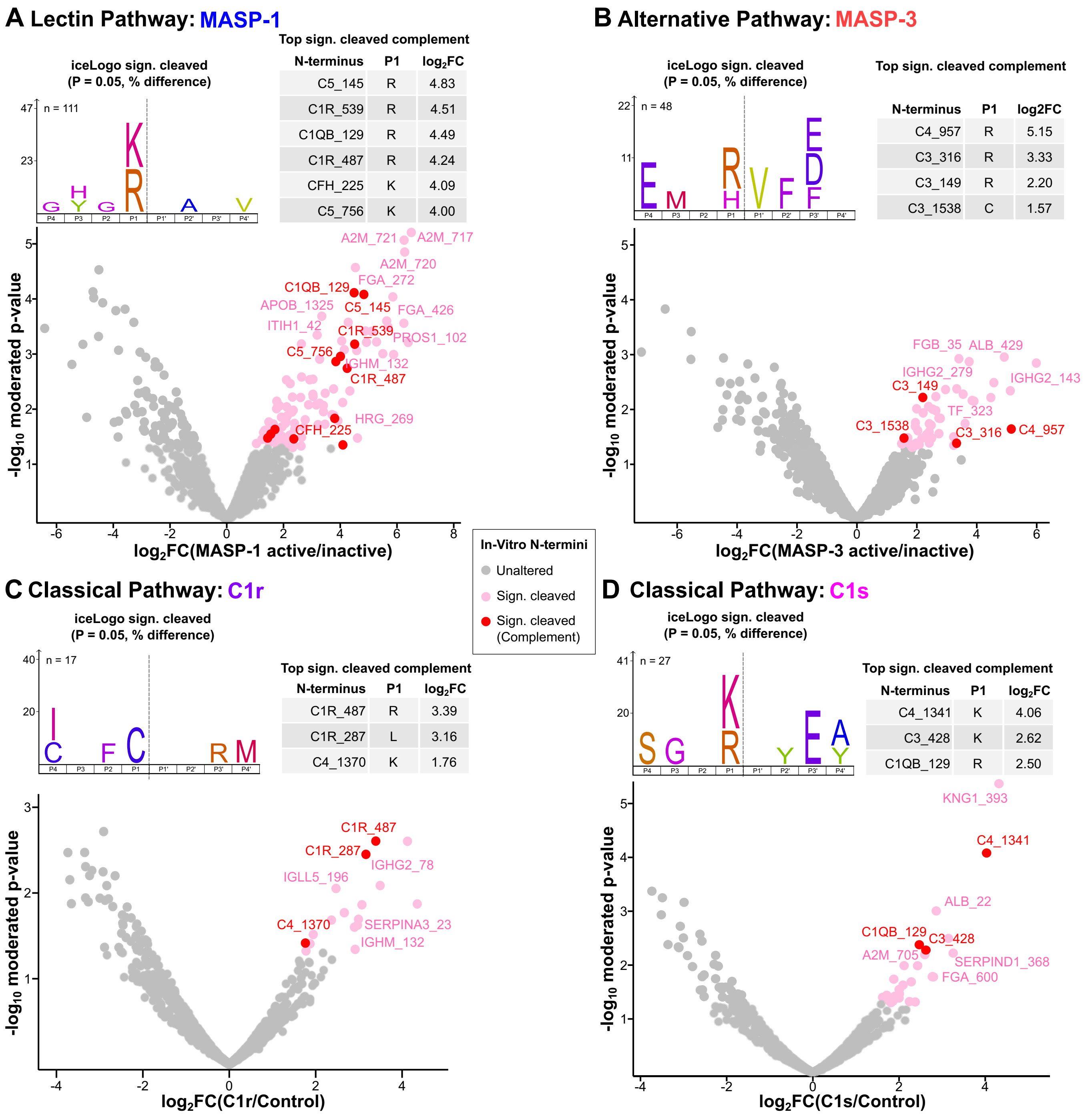

Figure EV2. In vitro profiling of major human complement initiating proteases, processed with trypsin.

Volcano plots and motifs of protease-induced proteolytic patterns for the lectin (MASP-1 (**A**)), alternative (MASP-3 (**B**)), and classical pathway (C1r/C1s (**C**, **D**)). After recombinant protease digestion (2 h, 37 °C) and dimethyl labeling, proteins were enzymatically digested using trypsin (Dataset EV10). Substrates were identified by differential abundance ($log_2FC > 1$ & limma moderated t-test p-value < 0.05) of active MASP-1/MASP-3 vs. inactive MASP-1/3 or active C1r/C1s vs. no protease control ($n = 4$ individual healthy plasma samples).

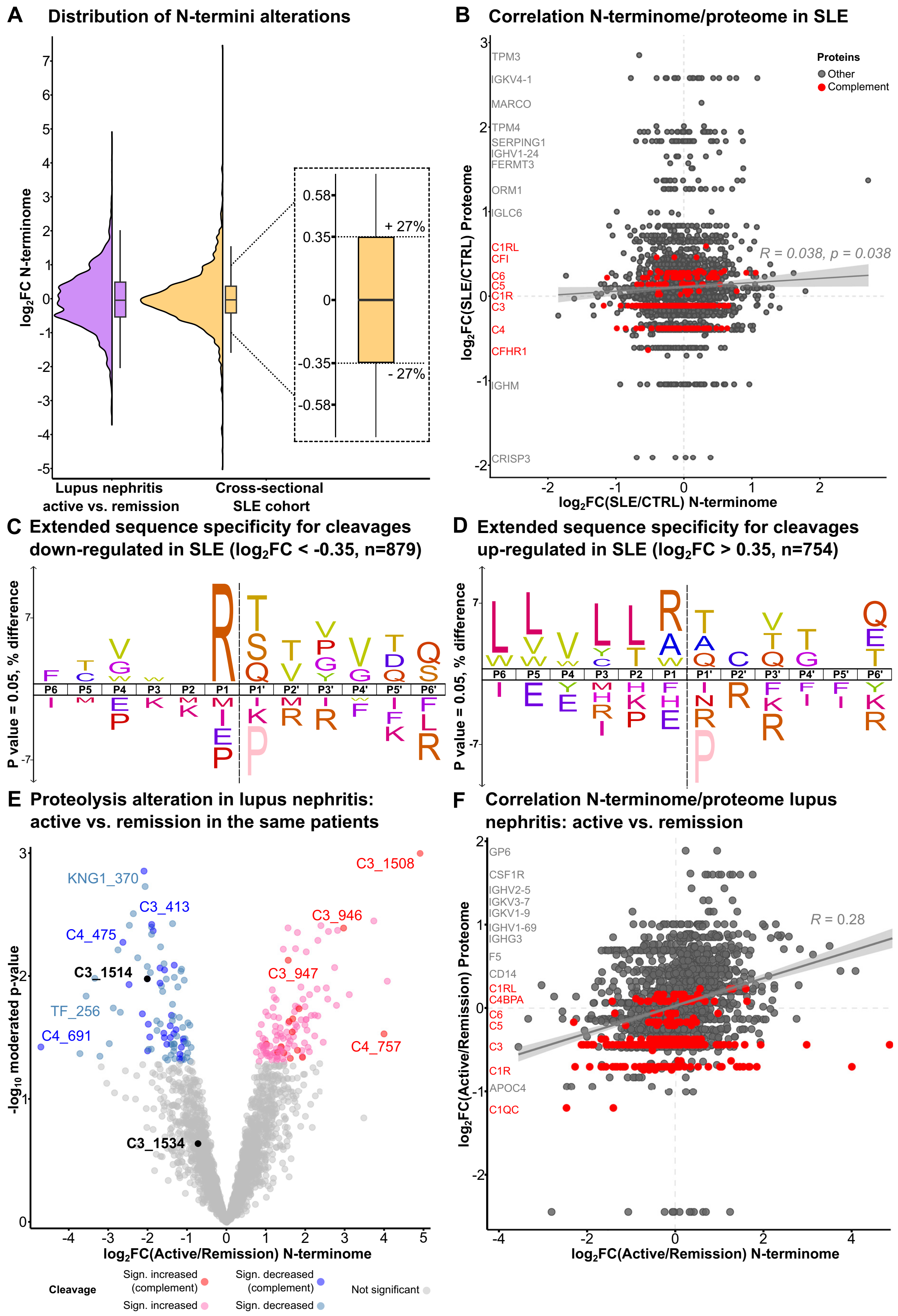
A Distribution of N-termini alterations
log2FC N-terminome
Lupus nephritis active vs. remission
Cross-sectional SLE cohort
+ 27%
- 27%
B Correlation N-terminome/proteome in SLE
Proteins
Other
Complement
R = 0.038, p = 0.038
TPM3
IGKV4-1
MARCO
TPM4
SERPING1
IGHV1-24
FERMT3
ORM1
IGLC6
C1RL
CFI
C6
C5
C1R
C3
C4
CFHR1
IGHM
CRISP3
log2FC(SLE/CTRL) Proteome
log2FC(SLE/CTRL) N-terminome
C Extended sequence specificity for cleavages down-regulated in SLE (log2FC < -0.35, n=879)
D Extended sequence specificity for cleavages up-regulated in SLE (log2FC > 0.35, n=754)
P value = 0.05, % difference
P6 P5 P4 P3 P2 P1 P1' P2' P3' P4' P5' P6'
E Proteolysis alteration in lupus nephritis: active vs. remission in the same patients
KNG1_370
C3_413
C4_475
C3_1514
TF_256
C4_691
C3_1534
C3_1508
C3_946
C3_947
C4_757
-log10 moderated p-value
log2FC(Active/Remission) N-terminome
Cleavage
Sign. increased (complement)
Sign. decreased (complement)
Not significant
Sign. increased
Sign. decreased
F Correlation N-terminome/proteome lupus nephritis: active vs. remission
R = 0.28
GP6
CSF1R
IGHV2-5
IGKV3-7
IGKV1-9
IGHV1-69
IGHG3
F5
CD14
C1RL
C4BPA
C6
C5
C3
C1R
APOC4
C1QC
log2FC(Active/Remission) Proteome
log2FC(Active/Remission) N-terminome

 © The Author(s)

Figure EV3. In vivo proteolysis alterations in SLE.

(**A**) Distribution of the N-termini alterations for the complete cross-sectional SLE cohort ($n = 166$ samples) and lupus nephritis trajectory ($n = 6$ patients). The first and third quartiles cut-off of log_2FC 0.35 (±27%) is defined as a filter regimen for the subsequent analysis steps of the SLE cohort and is displayed in the zoomed inset (boxplot line represents the median, whereas the box covers the first and third quartiles and the whiskers extend to the 1.5x Inter-Quartile-Range from the box). (**B**) A high variability of N-termini is detectable in complement proteins (red), which is lacking in the bulk proteome. The overall correlation for the N-terminome and proteome alterations was low (Pearson's R = 0.038). (**C**) Extended sequence specificity as visualized by iceLogo for the down-regulated N-termini in SLE ($log_2FC < -0.35$, $n = 879$ non-redundant N-terminal cleavage windows; *p*-value = chance of occurrence for every amino acid on every position < 0.05 cut-off used for generation) depicts an arginine-specific motif, mostly followed by glutamine, serine and threonine. (**D**) Extended sequence specificity of the up-regulated N-termini in SLE ($log_2FC > 0.35$, $n = 754$ non-redundant N-terminal cleavage windows, *p*-value < 0.05 cut-off used for generation) reveals a much more heterogeneous cleavage specificity with arginine in the center, but a remarkable leucine motif ahead of the cleavage site (P6, P5, P3 and P2). (**E**) In vivo proteolysis alteration in Lupus nephritis patients ($n = 6$ individual patients); the change in the N-termini of in active vs. remission states is displayed. Significantly decreased or increased N-termini ($|log_2FC| > 0.58$ & limma moderated t-test *p*-value < 0.05; Dataset EV9) are illustrated in pale blue and pink, whereas significantly altered complement N-termini are colored in blue and red, respectively. C3-LHF1 and an adjacent cleavage at D1534 are displayed in black. (**F**) The correlation between the N-terminome and proteome for the lupus nephritis patients was substantially higher (Pearson's R = 0.28). A general increase in the proteome abundance of complement proteins can be observed in the active state, whereas the proteolytic processing appears to differ.

© The Author(s)

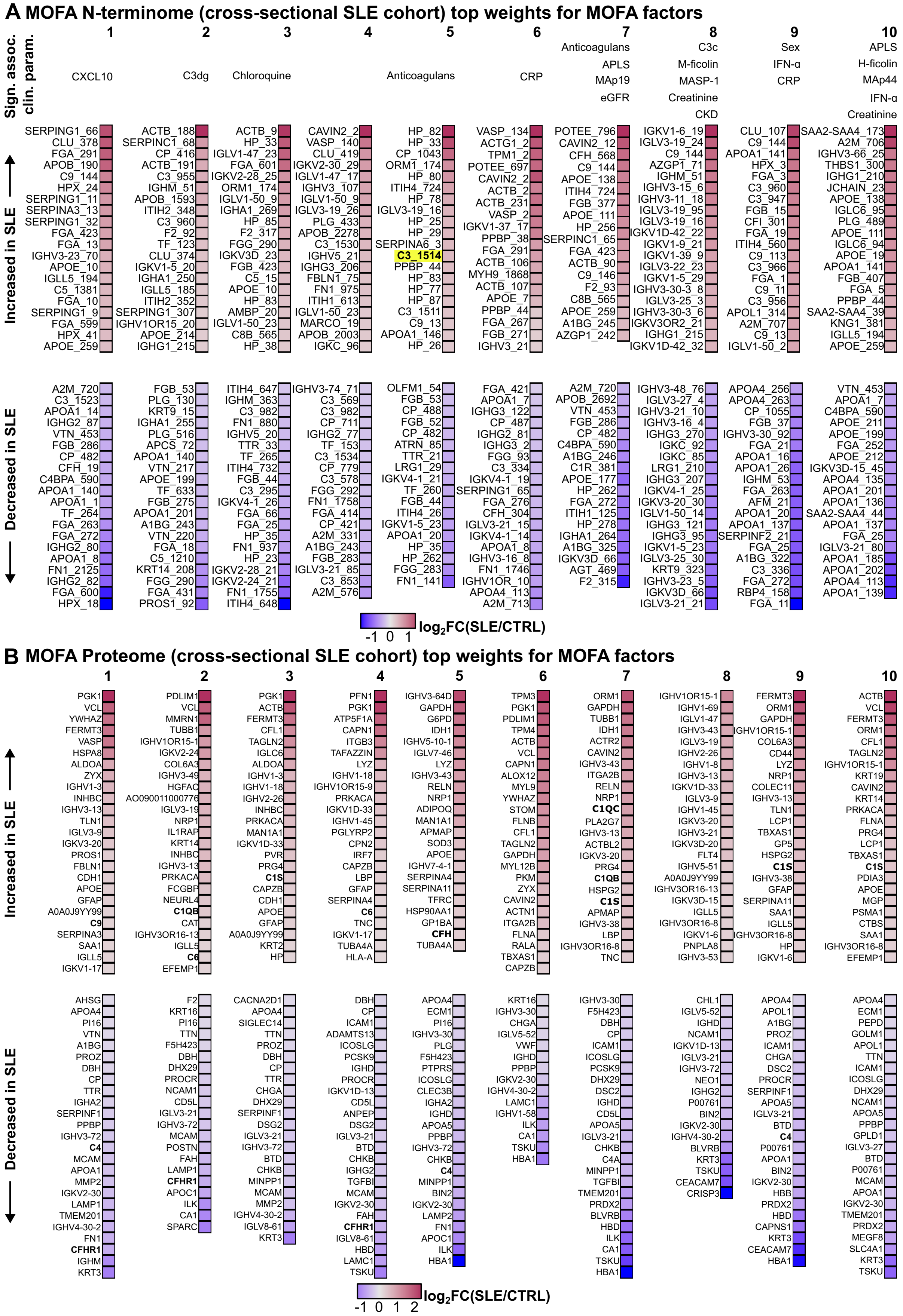
A MOFA N-terminome (cross-sectional SLE cohort) top weights for MOFA factors
Sign. assoc. clin. param.
1 CXCL10
2 C3dg
3 Chloroquine
4
5 Anticoagulans
6 CRP
7 Anticoagulans APLS MAp19 eGFR
8 C3c M-ficolin MASP-1 Creatinine CKD
9 Sex IFN-α CRP
10 APLS H-ficolin MAp44 IFN-α Creatinine
Increased in SLE
Decreased in SLE
log2FC(SLE/CTRL)
-1 0 1
B MOFA Proteome (cross-sectional SLE cohort) top weights for MOFA factors
1 2 3 4 5 6 7 8 9 10
Increased in SLE
Decreased in SLE
log2FC(SLE/CTRL)
-1 0 1 2

 © The Author(s)

Figure EV4. Top N-terminome & proteome weights for all MOFA factors.

(**A**) N-terminome-based top positive weights with the most substantial influence on each of the ten MOFA factors were assessed in the cross-sectional SLE patient data, filtered for a minimal log_2FC of 0.35 (27% alteration, cf. Fig. EV3A), and sorted by increased (red) or decreased (blue) abundance in SLE. The candidate N-terminus C3_1534 (C3-LHF1) is prominently present in factor 5 (bold, highlighted in yellow). (**B**) Proteome-based top positive weights in the cross-sectional SLE patient data are given.

© The Author(s)

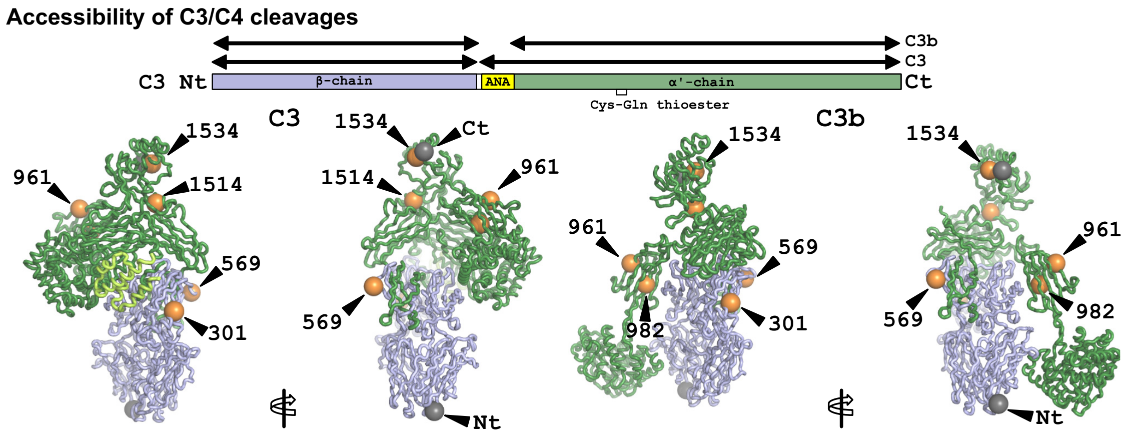

Figure EV5. Complement C3 N-termini mapping.

Mapping MOFA-selected C3 cleavages on PDB structures demonstrates accessibility, especially for the C3-LHF1 cleavage (pos. 1514) in C3 and C3b.

 © The Author(s)

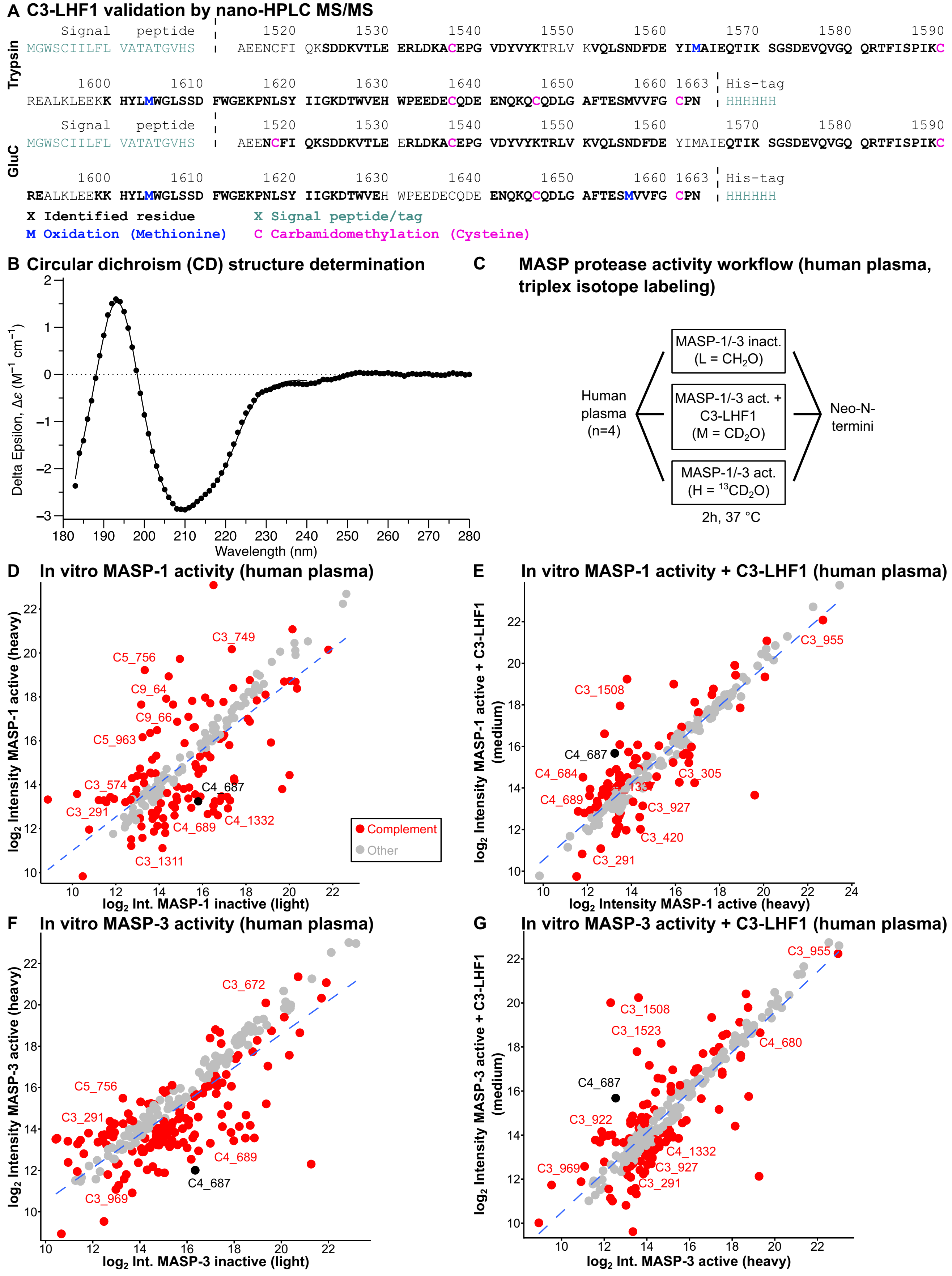
A C3-LHF1 validation by nano-HPLC MS/MS
Trypsin
Signal peptide
1520 1530 1540 1550 1560 1570 1580 1590
MGWSCIILFL VATATGVHS AEENCFI QKSDDKVTLE ERLDKACEPG VDYVYKTRLV KVQLSNDFDE YIMAIEQTIK SGSDEVQVGQ QRTFISPIKC
1600 1610 1620 1630 1640 1650 1660 1663 His-tag
REALKLEEKK HYLMWGLSSD FWGEKPNLSY IIGKDTWVEH WPEEDECQDE ENQKQCQDLG AFTESMVVFG CPN HHHHHH
GluC
Signal peptide
1520 1530 1540 1550 1560 1570 1580 1590
MGWSCIILFL VATATGVHS AEENCFI QKSDDKVTLE ERLDKACEPG VDYVYKTRLV KVQLSNDFDE YIMAIEQTIK SGSDEVQVGQ QRTFISPIKC
1600 1610 1620 1630 1640 1650 1660 1663 His-tag
REALKLEEKK HYLMWGLSSD FWGEKPNLSY IIGKDTWVEH WPEEDECQDE ENQKQCQDLG AFTESMVVFG CPN HHHHHH
X Identified residue
X Signal peptide/tag
M Oxidation (Methionine)
C Carbamidomethylation (Cysteine)
B Circular dichroism (CD) structure determination
Delta Epsilon, Δε (M⁻¹ cm⁻¹)
Wavelength (nm)
C MASP protease activity workflow (human plasma, triplex isotope labeling)
Human plasma (n=4)
MASP-1/-3 inact. (L = CH2O)
MASP-1/-3 act. + C3-LHF1 (M = CD2O)
MASP-1/-3 act. (H = 13CD2O)
2h, 37 °C
Neo-N-termini
D In vitro MASP-1 activity (human plasma)
log2 Intensity MASP-1 active (heavy)
log2 Int. MASP-1 inactive (light)
C3_749
C5_756
C9_64
C9_66
C5_963
C3_574
C4_687
C3_291
C4_689
C4_1332
C3_1311
Complement
Other
E In vitro MASP-1 activity + C3-LHF1 (human plasma)
log2 Intensity MASP-1 active + C3-LHF1 (medium)
log2 Intensity MASP-1 active (heavy)
C3_955
C3_1508
C4_687
C4_684
C3_305
C4_689
C3_927
C3_420
C3_291
F In vitro MASP-3 activity (human plasma)
log2 Intensity MASP-3 active (heavy)
log2 Int. MASP-3 inactive (light)
C3_672
C5_756
C3_291
C4_689
C4_687
C3_969
G In vitro MASP-3 activity + C3-LHF1 (human plasma)
log2 Intensity MASP-3 active + C3-LHF1 (medium)
log2 Int. MASP-3 active (heavy)
C3_955
C3_1508
C3_1523
C4_680
C4_687
C3_922
C4_1332
C3_969
C3_927
C3_291

Figure EV6. Sequence and structural characterization of recombinant C3-LHF1.

(**A**) Recombinant C3-LHF1 was processed with the two different digestion enzymes, trypsin, and GluC, and subsequently analyzed by nano-LC-MS/MS. Almost full sequence coverage (bold) was achieved in database searches against the host CHO proteome for heterologous protein production, supplemented with the C3-LHF1 sequence. (**B**) The recombinant LHF-1 protein sample was analyzed using Synchrotron Radiation Circular Dichroism (SRCD) and compared with the crystal structure of the C345c domain within the structure of full-length complement C3 (PDB entry 2A73). The fit of the spectrum resulted in an estimation of 17 ± 3% α-helix, 25 ± 5% β-sheet, 13 ± 4% turns and 39 ± 6% other unordered structures as the secondary structure content. This compares well with the secondary structure content percentages of the crystal structure of the C345c domain of complement C3, obtained via DSSP which is ~28% α-helix, ~33% β-sheet and ~39% other structures. (**C**) Workflow for determining the MASP-1/3 protease activity in absence/presence of 20 µM C3-LHF1 in a triplex isotope labeling scheme on human EDTA-plasma, where samples after protease incubation were pooled before SHUNTER N-termini enrichment. (**D–G**) In vitro protease activity was measured in a triplex isotope labeling on human plasma incubated with inactive MASP-1/3 (subsequently labeled with light formaldehyde CH_2O), active MASP-1/3 (heavy formaldehyde $^{13}CD_2O$) and active MASP-1/3 + C3-LHF1 (medium formaldehyde CD_2O, $n = 4$ individual plasma samples). Complement cleavages (marked in red or black for C4_687) significantly deviating in log_2-transformed N-termini intensity between compared conditions, active protease vs. inactive controls (**D**, **F**) or active protease vs. active protease + 20 µM C3-LHF1 (**E**, **G**) are labeled with residue number.

© The Author(s)

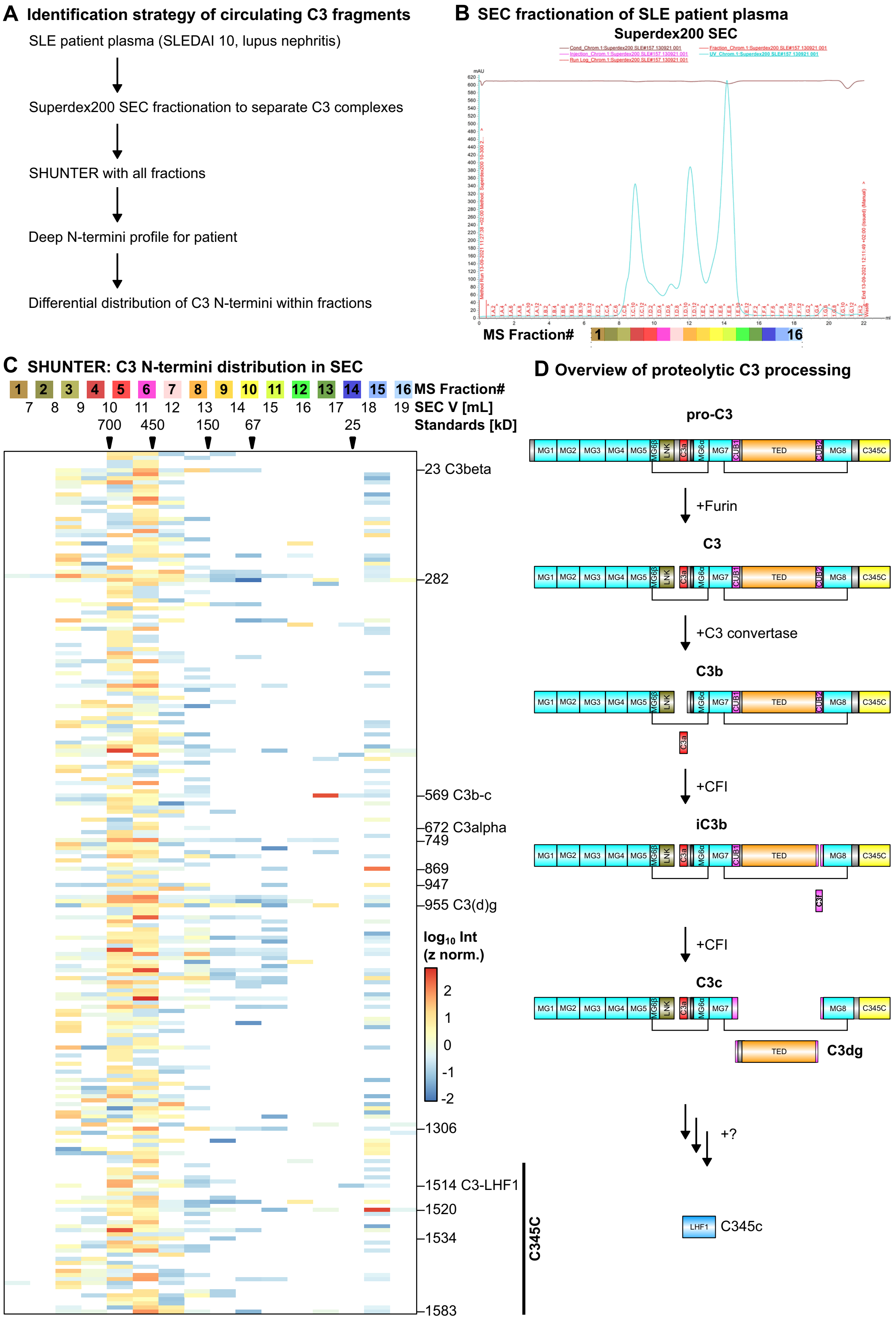
A Identification strategy of circulating C3 fragments
SLE patient plasma (SLEDAI 10, lupus nephritis)
Superdex200 SEC fractionation to separate C3 complexes
SHUNTER with all fractions
Deep N-termini profile for patient
Differential distribution of C3 N-termini within fractions
B SEC fractionation of SLE patient plasma
Superdex200 SEC
MS Fraction#
1
16
C SHUNTER: C3 N-termini distribution in SEC
1 2 3 4 5 6 7 8 9 10 11 12 13 14 15 16 MS Fraction#
7 8 9 10 11 12 13 14 15 16 17 18 19 SEC V [mL]
700 450 150 67 25 Standards [kD]
23 C3beta
282
569 C3b-c
672 C3alpha
749
869
947
955 C3(d)g
log10 Int (z norm.)
2
1
0
-1
-2
1306
1514 C3-LHF1
1520
1534
1583
C345C
D Overview of proteolytic C3 processing
pro-C3
MG1 MG2 MG3 MG4 MG5 MG6β LNK C3a MG6α MG7 CUB1 TED CUB2 MG8 C345C
+Furin
C3
+C3 convertase
C3b
C3a
+CFI
iC3b
C3f
+CFI
C3c
C3dg
+?
LHF1 C345c

Figure EV7. Size exclusion chromatography of SLE patient plasma to identify fragment elution behavior.

(**A**) Strategy to identify circulating complement factors by using size-exclusion chromatography (SEC). (**B**) SEC separation of a severely diseased SLE patient plasma sample (SLEDAI score of 10, LN) yielded sixteen combined 1 mL fractions (1–16), subjected to SHUNTER N-termini enrichment. (**C**) Heatmap for the overview of the complement C3 N-termini distribution in the SEC MS fractions. The distribution is z-score normalized for each N-terminus, yielding red for a hotspot of the corresponding N-terminus, whereas, in blue labeled fractions, the corresponding N-terminus is only weakly present. C3-LHF1 displays an intermediate distribution profile between C3b (C3_23) and C3(d)g (C3_955) fragments. (**D**) Schematic overview of the proteolytic processing for complement factor C3.

 © The Author(s)
